# Lifetime changes in body maps investigated with music

**DOI:** 10.1515/tnsci-2025-0407

**Published:** 2026-10-23

**Authors:** Masaki Tanaka, Tatsuya Daikoku

**Affiliations:** Graduate School of Information Science and Technology, The University of Tokyo, 7-3-1 Hongo, Bunkyo-ku, Tokyo, 113-8656, Japan

**Keywords:** prediction, body map, emotion, lifetime, interoception

## Abstract

This study investigates how musical prediction mechanisms – specifically uncertainty and surprise – shape emotions and bodily sensations across the lifespan. To examine this, we conducted online experiments with 784 participants aged 10 to 88, using eight chord progressions derived from computational predictive models to assess emotional responses and bodily sensation maps. The results showed that overall bodily sensation intensity peaked in the 20s to 30s and then declined with age, while the relative proportion of head-centered sensations increased significantly across the lifespan. In contrast, subjective valence ratings remained stable across the lifespan, whereas arousal levels increased with age and showed a strong correlation with predictable musical structures. These findings suggest an age-related shift toward top-down cognitive prediction alongside preserved emotional appraisal, while supporting the relationship between top-down processing shaped by accumulated experience and the predictability of music, as indicated by previous research. By accounting for the unique physical and emotional characteristics of each age group, this study may support the development of prediction- and music-based interventions aimed at understanding generation-specific emotional and interoceptive responses to music.

## Introduction

Music is a universal medium that plays a vital role in shaping human emotional experiences [1]. To understand how music enhances affective experience across the lifespan, it is essential to uncover the underlying cognitive and interoceptive mechanisms – particularly musical prediction – that transform auditory patterns into physical and emotional experiences resonance.

Previous research has shown that music is linked to various psychological elements, including emotion regulation, cognitive processing, and social bonding, and plays a significant role in maintaining and altering states such as mood, emotion, arousal levels, and reminiscence [2], [3], [4]. Music is appreciated by people of all ages, from children to the elderly and adults alike. Sloboda and colleagues [5] found that music listening in adults’ daily lives significantly influences emotions, including reminiscence and mood. In younger individuals, music has been studied as a tool for emotional regulation and cognitive adjustment [6]. Additionally, Laukka [7] found that listening to music among older adults frequently elicits positive emotions, contributing to positive affective experiences.

Numerous studies have demonstrated that music significantly affects bodily sensations related to stress, ultimately influencing both physiological and psychological outcomes [8], [9], [10], [11]. Recently, research has increasingly focused on bodily responses, particularly interoceptive sensations, to better understand the relationship between music and emotion [12]. A recent study by Putkinen and colleagues [13] used bodily sensation maps to visualize these responses [14], 15] and found that music can evoke strong subjective bodily emotions that correlate with the emotional experiences it elicits. These findings support models that highlight the importance of interoceptive sensations in the experience of emotions [16], 17]. Additionally, our previous study [18] identified specific body regions associated with emotional responses to musical prediction. Our findings revealed that positive emotions evoked by predictable patterns are strongly felt in the chest, whereas negative emotions, such as anxiety and confusion arising from prediction error, are linked to sensations in the head. Based on these foundational studies, specific body regions can be categorized by their functional roles in music processing: the head region primarily reflects top-down cognitive processing and localized auditory prediction error [18]; the cardiac (chest) region is closely associated with emotional arousal and sympathetic autonomic reactivity [14], 18]; and the abdominal region is linked to visceral interoceptive awareness and valence (“gut feelings”) [16], 18].

However, previous studies on music, emotions, and bodily responses have primarily focused on adults, accounting for various individual and cultural differences. While music is known to enhance emotional and bodily responses across age groups, research examining the relationship between music and these aspects across generations, including school-age children and older adults, remains limited.

Therefore, we aim to clarify how musical prediction influences interoception and bodily sensations across the lifespan. We also seek to identify the specific musical factors that enhance positive emotions and affective engagement. In particular, we examined how bodily sensations evoked by musical prediction – defined by the statistical uncertainty of the musical progression and the surprise (prediction error) of specific chords – relate to emotional responses in terms of valence and arousal. To achieve this, we conducted online experiments that included participants aged 10 to 88, focusing on body mapping and emotional assessments using music based on eight types of four-chord progressions. To systematically manipulate musical expectation, these chord progressions were categorized into four predictive states using the Information Dynamics of Music (IDyOM) framework, based on two dimensions: Surprise (High vs. Low) and Uncertainty (High vs. Low). Specifically, we formulated explicit directional hypotheses for the response patterns across the chord progressions: sequence types characterized by high surprise and/or high uncertainty (e.g., sHuH-sHuH, sLuL-sHuH) were expected to elicit heightened physiological arousal and stronger, head- and cardiac-centered bodily sensations driven by prediction errors. Conversely, sequence types characterized by low surprise and low uncertainty (e.g., sLuL-sLuL) were expected to evoke baseline bodily sensations aligned with emotional stability, relaxation, and higher positive valence. These chord progressions consider aspects of musical uncertainty, prediction errors, and temporal dynamics. The BRECVEMA framework proposed by Juslin [19] and the research by Vuust and colleagues [20] indicate that predictions about music significantly influence its emotional impact. Furthermore, our previous studies have shown that these chord progressions are associated with various emotional components of music [18], [21], [22], [23].

Furthermore, given potential age-related shifts in interoceptive sensitivity [18] and predictive cognitive processing [18], we hypothesized that bodily sensation maps would exhibit region-specific developmental trajectories across the lifespan. Specifically, we expected interoceptive and autonomic regions (chest/cardiac and abdominal areas) to follow an inverted U-shaped pattern – increasing through adolescence, peaking in young adulthood (20s–30s), and gradually declining in older adulthood due to reduced peripheral interoceptive sensitivity. In contrast, we hypothesized that head-centered sensations, driven by top-down cognitive and predictive mechanisms, would show a distinct developmental trajectory less susceptible to peripheral sensory decline.

## Materials and methods

The experimental paradigm and auditory stimuli are summarized in Figure 1. After listening to eight distinct chord progressions that varied in surprise and uncertainty, participants completed a body-mapping task and emotional evaluations (valence and arousal).

**Figure 1:**
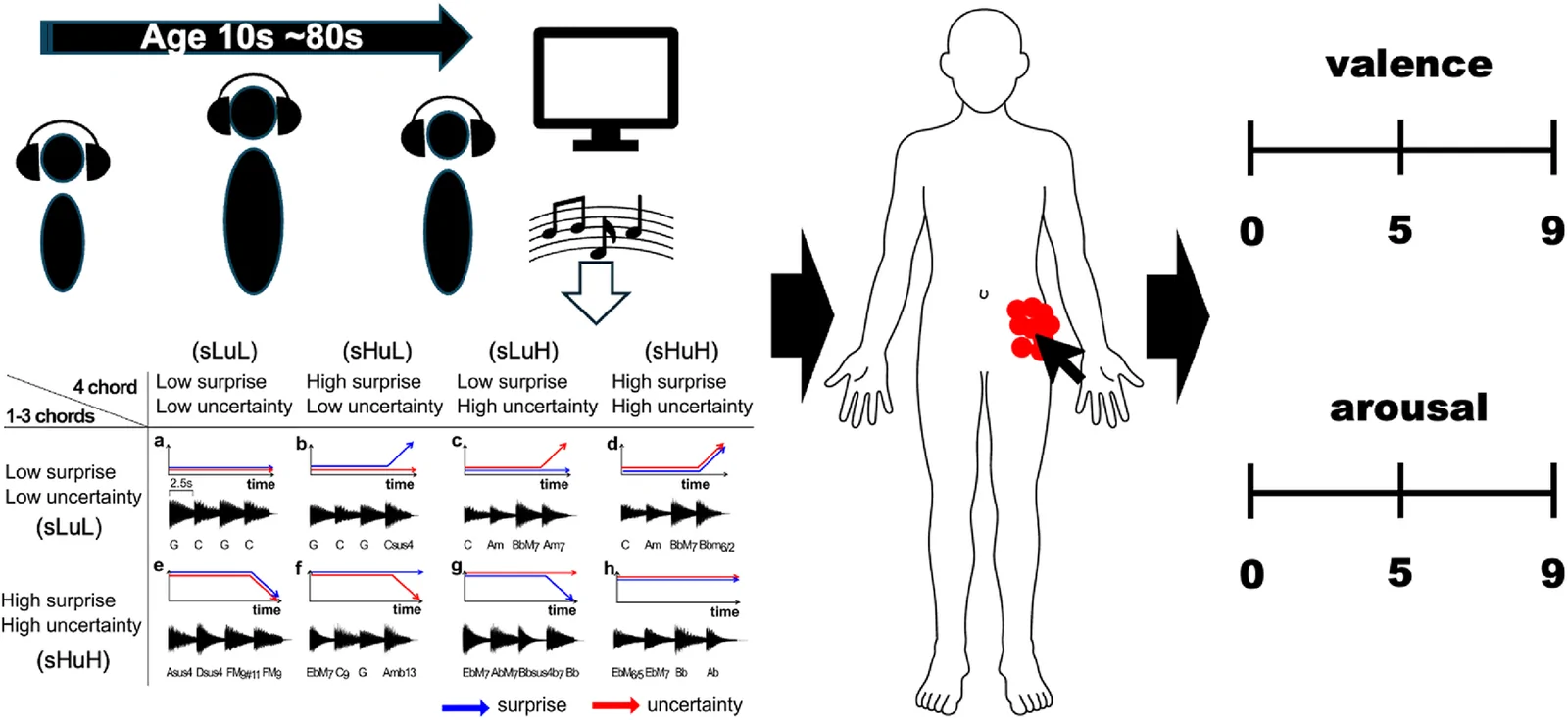
The experimental paradigm and sound stimuli. Below are 8 types of chord progressions. The blue and red arrows indicate surprise and uncertainty values, respectively.

### Participants

The present study involved conducting bodily sensation mapping tests along with emotional judgments for each of the eight types of 4-chord progressions (for details, see [21]). A total of 784 Japanese participants, aged 10–88 years (mean age ± SD=47.42 ± 21.34; 372 females, 411 males, 1 unspecified), participated in the study. Participants were grouped into eight 10-year age bins (Figure S1): 10s (n=101, 51 females), 20s (n=104, 52 females), 30s (n=103, 50 females), 40s (n=105, 52 females), 50s (n=106, 54 females), 60s (n=105, 55 females), 70s (n=102, 50 females), and 80s (n=58, 8 females). All participants reported no history of neurological or audiological disorders and did not possess absolute pitch. The experiment was conducted in accordance with the guidelines of the Declaration of Helsinki and received approval from the Ethics Committee of The University of Tokyo (Approval No. UT IST RE 230601). All participants provided informed consent and conducted the experiments on a PC.

## Materials

The experimental paradigm was developed using the Gorilla Experiment Builder, a cloud-based research platform that allows for the online deployment of behavioral experiments. Each participant listened to eight different four-chord progressions, with each chord lasting 500 ms (total sequence duration=2,000 ms). The audio was played at a sampling rate of 44.1 kHz and in 32-bit format, using General MIDI’s Electric Piano 1 sound. To ensure consistent loudness perception across all participants, the amplitudes of the chords were adjusted according to equal-loudness contours.

This study focuses specifically on the emotional effects of local musical structures, drawing inspiration from neuroscientific findings by Koelsch [24] and Cheung et al. [25], which demonstrate that local features such as disharmony and unexpectedness can reliably elicit emotional responses. To quantitatively manipulate musical expectancy, we used a variable-order Markov n-gram statistical learning model [26] within the IDyOM framework, consistent with our prior experimental design [21]. The model was pre-trained on the McGill Billboard corpus [27], an expert-annotated database of 890 Western pop songs, to extract conditional transition probabilities between adjacent chords and to compute Shannon information content and entropy [28].

For any given chord *x*_*i*_ occurring after a context sequence *x*_1:*i*-1_, two information-theoretic metrics were calculated:

Surprisal (Information Content, *I*): Quantifying retrospective prediction error or surprise associated with chord *x*_*i*_:

$$I\left(x_{i}|x_{1:i-1}\right)=-\log_{2}\;P\left(x_{i}|x_{1:i-1}\right)$$

Uncertainty (Entropy, *H*): Quantifying prospective uncertainty over the probability distribution of all possible upcoming chords X at position *i*:

$$H\left(X_{i}|x_{1:i-1}\right)=-\underset{x\in X}{\sum }P\left(x|x_{1:i-1}\right)\log_{2}\;P\left(x|x_{1:i-1}\right)$$

– To classify chords into distinct high-vs. low-predictability states, empirical distributions of *I* and *H* values were derived across all candidate chord transitions in the corpus-trained model. Thresholds for “High” and “Low” statistical states were defined as follows:
– Low (L) condition: Values in the bottom 20th percentile (≤20th percentile) of the corpus-wide empirical distribution for *I* or *H.*
– High (H) condition: Values in the top 20th percentile (>80th percentile) of the corpus-wide empirical distribution for *I* or *H.*
– Values within the middle range (21st–79th percentiles) were excluded to maximize signal contrast across predictability categories.

Using these criteria, a total of 92 distinct four-chord progressions were synthesized and categorized into eight experimental conditions. In each sequence, the context chords (Chords 1–3) maintained a consistent statistical profile – either uniformly low surprise and low uncertainty (sLuL context) or uniformly high surprise and high uncertainty (sHuH context). The target chord (Chord 4) was manipulated independently across two levels of Surprisal (High/Low) and two levels of Uncertainty (High/Low):

– sLuL-sLuL (14 progressions): Context (Low *I*, Low *H*) → Target (Low *I*, Low *H*)
– sLuL-sHuL (14 progressions): Context (Low *I*, Low *H*) → Target (High *I*, Low *H*)
– sLuL-sLuH (12 progressions): Context (Low *I*, Low *H*) → Target (Low *I*, High *H*)
– sLuL-sHuH (8 progressions): Context (Low *I*, Low *H*) →Target (High *I*, High *H*)
– sHuH-sLuL (18 progressions): Context (High *I*, High *H*) → Target (Low *I*, Low *H*)
– sHuH-sHuL (14 progressions): Context (High *I*, High *H*) → Target (High *I*, Low *H*)
– sHuH-sLuH (3 progressions): Context (High *I*, High *H*) → Target (Low *I*, High *H*)
– sHuH-sHuH (9 progressions): Context (High *I*, High *H*) → Target (High *I*, High *H*)

During the experiment, each participant was presented with eight chord progressions in randomized order, with exactly one representative progression selected from each of the eight condition categories, ensuring equal exposure to all statistical conditions while preventing participant fatigue.

### Procedures

Participants were presented with eight different chord progressions in a random order. Before the main experimental trials, participants completed a preliminary audio verification task to set a comfortable listening volume, verify proper sound playback, and ensure understanding of the instructions. During this test, participants clicked a play button to listen to spoken audio guidance and then selected the corresponding number spoken in the audio (see Figure S2A in the Supplementary Material).

Each main experimental trial consisted of two sequential tasks: a bodily mapping task followed by a subjective rating task. On the initial screen of each trial, an audio playback button (“Play”) and a human body outline were displayed alongside the explicit instruction: “Please click, within 3 s, on each body region where you feel sensations in response to the sound. There is no limit on the number of clicks*”* (presented in Japanese; see Figure S2B in the Supplementary Material). Participants initiated audio playback by clicking the button to listen to the chord progression and then clicked the body illustration within 3 s. This instruction ensured that participants were free to click any body location as many times as applicable, without restriction, capturing both the spatial distribution and the subjective magnitude of bodily sensations. Advancing to the next trial without making any clicks was also permitted if no bodily sensation was reported. This bodily mapping method, based on self-reported clicks, has demonstrated validity in previous studies using similar approaches [14], 18], 29].

Immediately after completing the bodily mapping task for each chord progression, participants moved to a second screen to evaluate their subjective impressions of the progression using 9-point Likert scales across four dimensions: Valence (1: Unpleasant to 9: Pleasant), Arousal (1: Calm to 9: Active), Creativity (1: Uncreative to 9: Creative), and Aesthetic Value (1: Unbeautiful to 9: Beautiful). A score of 5 indicated a neutral response (see Figure S2C in the Supplementary Material). To address the primary research questions of the present study, only Valence and Arousal ratings were included in the statistical analysis, while Creativity and Aesthetic Value ratings were excluded from the main analysis.

### Quantification and statistical analysis

Using the coordinate data of x and y from the body mapping test, we extracted the total number of clicks at three regions of interest (ROIs): the cardiac, abdominal, and head areas for each participant. The raw x and y coordinate data (see Figure S3 in the Supplementary Material) were downsampled by a factor of 40. The body image template used measured 871 pixels in width and 1,920 pixels in height. The body topography figures (Figure 2) were generated using Matlab (2022b) by interpolating the x and y coordinates in a mesh grid format, employing a color map to represent the neighboring points. To prepare the input data for these maps, we computed the average click frequency at each (x, y) coordinate across all participants. To address potential confounds related to overall click propensity and age-related psychomotor slowing, we performed two complementary evaluations: (i) we evaluated whether response counts were time-limited in practice by examining trial-level click frequency distributions across all experimental trials (15,698 trials in total), and (ii) we evaluated relative ROI proportions by dividing region-specific click counts by each participant’s total click count. We fit linear mixed-effects models (LMMs) to these relative proportions, with Age and Chord progression as fixed effects and participant ID as a random intercept. To assess the empirical sensitivity of our sample size (n=784 participants, yielding n=18,816 repeated-measures observations), we conducted a sensitivity power analysis in MATLAB. At *α*=0.05 and 80 % statistical power (1 − *β*=0.80), our linear model framework had sufficient sensitivity to detect small effect sizes at the participant level (Cohen’s *f*=0.1002, *η*_*p*_^*2*^=0.0099) and very small effect sizes at the repeated-measures observation level (Cohen’s *f*=0.0204, *η*_*p*_^*2*^=0.0004), confirming high empirical sensitivity for detecting both main effects and subtle interactions.

**Figure 2:**
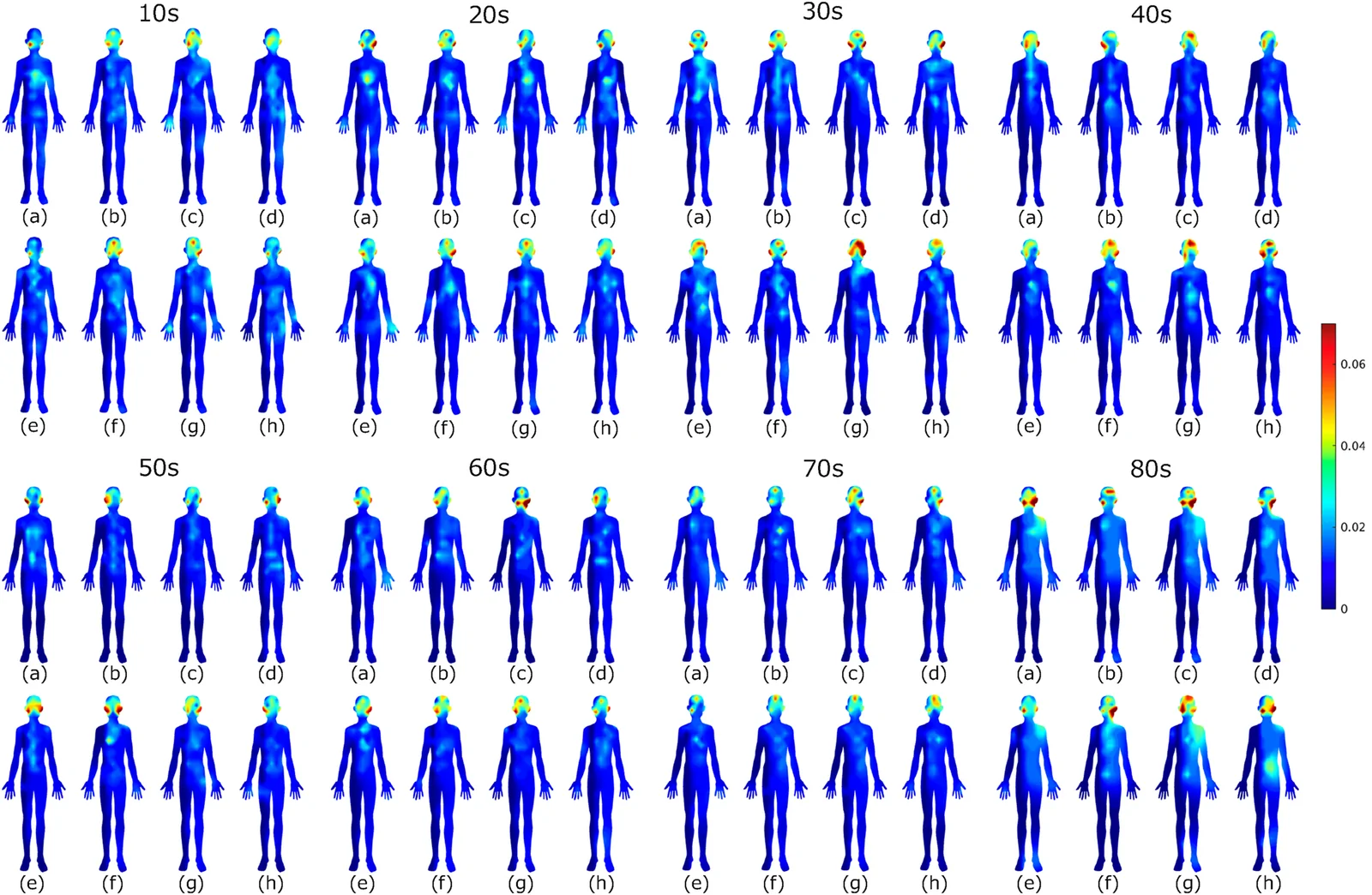
Raw body topography for chord progressions for each age group. The blue-to-red gradients represent unadjusted raw click counts, ranging from low to high response density. Across all chord progressions, absolute bodily response volume peaked in the 30s age group and declined with increasing age. (a): sLuL-sLuL, (b): sLuL-sHuL, (c): sLuL-sLuH, (d): sLuL-sHuH, (e): sHuH-sLuL, (f): sHuH-sHuL, (g): sHuH-sLuH, (h): sHuH-sHuH. High-resolution, enlarged body-topography maps for each age group and condition are publicly available on the OSF repository (https://osf.io/wzvqj/).

To account for repeated measures and individual variance across participants, we conducted statistical analyses in jamovi (version 2.7) and MATLAB (R2022b). We set statistical significance at p<0.05 for all tests.

For body map sensation intensity (click counts), we fitted Generalized Linear Mixed-Effects Models (GLMMs) with Poisson or negative binomial distributions (to account for count data). Fixed effects included Age (either as a continuous predictor or in 10-year age bins), body region (Cardiac, Abdominal, Head), Chord Prediction Category (e.g., sLuL, sHuH), and their two- and three-way interactions. Random intercepts were included for Participants and Chord Items to account for random variation across individuals and across specific musical stimuli:

$$C\text{lick Count }∼\text{ Age}\times R\text{egion}\times \text{Chord Category}+\left(1\;\left|\text{ Participant})+(1\;\right|\text{Item}\right)$$

To account for potential confounding effects of task repetition and temporal dependencies across consecutive trials (e.g., practice, fatigue, or serial dependence), we also specified an additional Linear Mixed-Effects Model (LMM). This sensitivity model evaluated bodily sensation proportions while controlling for trial presentation order (Trial Order) and the preceding trial’s response (Lag1 Proportion) as continuous covariates, in addition to the primary fixed factors (Supplementary Tables S1 and S2).

For subjective valence and arousal ratings, we fitted Linear Mixed-Effects Models (LMMs) with the same fixed and random factor structure. Sum-of-squares Type III ANOVA tables were computed to test omnibus main effects and interactions. Post hoc comparisons and simple main effect analyses (e.g., age slopes within specific body regions or chord conditions) were conducted using estimated marginal means (emmeans) with Holm-adjusted p-values (or False Discovery Rate, FDR, where applicable) to control for multiple comparisons. For age-group comparisons, post hoc pairwise evaluations were conducted using Wilcoxon rank-sum tests with false discovery rate (FDR) correction, and effect sizes were reported as Cohen’s d with 95 % confidence intervals (95 % CI). Sensitivity analyses compared models with Age as a continuous predictor to those with Age in 10-year categorical bins to ensure that grouping did not introduce analytical bias. To further evaluate non-significant main effects and interactions, we conducted Bayesian model comparisons using Bayesian Information Criterion (BIC) approximations for linear mixed-effects models fitted via Maximum Likelihood (ML). Bayes factors (*BF*_01_ and *BF*_10_) were calculated *BF*_01_=exp((*BIC*_*H*1_−*BIC*_*H*0_)/2) and *BF*_10_=1/*BF*_01_. According to Jeffreys’ benchmarks, *BF*_*01*_>10 was interpreted as strong evidence in favor of the null hypothesis (H_0_), allowing us to distinguish between a true absence of an effect and statistical insensitivity.

## Results

### Relationship between age and bodily sensations in each chord progression

To directly test our explicit hypotheses about the eight chord progressions, we evaluated emotional and bodily responses across the four predictive states (high/low surprise × high/low uncertainty). As expected, progressions with high surprise and uncertainty (e.g., sHuH) yielded higher arousal ratings and stronger localized bodily sensations, whereas low-surprise/low-uncertainty progressions (e.g., sLuL) were associated with baseline sensations and higher positive valence.

The eight chord-progression conditions represented four predictive states defined by the IDyOM framework: High/Low Surprise × High/Low Uncertainty. To evaluate how musical predictive processing influences somatic responses across the lifespan, we analyzed the spatial distribution of reported bodily sensations across participants (n=784). Bodily sensations were evaluated in specific regions of interest (ROIs): interoceptive regions (cardiac and abdominal areas) and the head region, where top-down cognitive and auditory prediction errors are localized. To account for potential individual differences in overall clicking frequency (e.g., age-related psychomotor slowing), we defined the primary dependent variable as the proportion of clicks within each ROI relative to total body clicks per trial.

Figure 2 and Supplementary Figure S3 show grand-average bodily sensation topographies and raw click coordinate distributions across age groups and chord progressions. All statistical analysis results, including descriptive statistics, are available on the Open Science Framework (https://osf.io/wzvqj/).

To test our primary hypothesis about age-related changes in bodily sensations at the individual level (n=784), while controlling for trial sequence (Trial Order) and serial dependencies (Lag1 Proportion), we fitted a Linear Mixed-Effects Model (LMM; Supplementary Table S1). The omnibus test revealed significant main effects of Region (*F*(2, 16,414)=648.59, p<0.001) and Age (*F*(1, 16,414)=7.34, p=0.007), as well as significant two-way interactions between Chord and Region (*F*(14, 16,414)=6.79, p<0.001) and between Region and Age (*F*(2, 16,414)=11.15, p<0.001; Figure 3, Supplementary Table S1).

**Figure 3:**
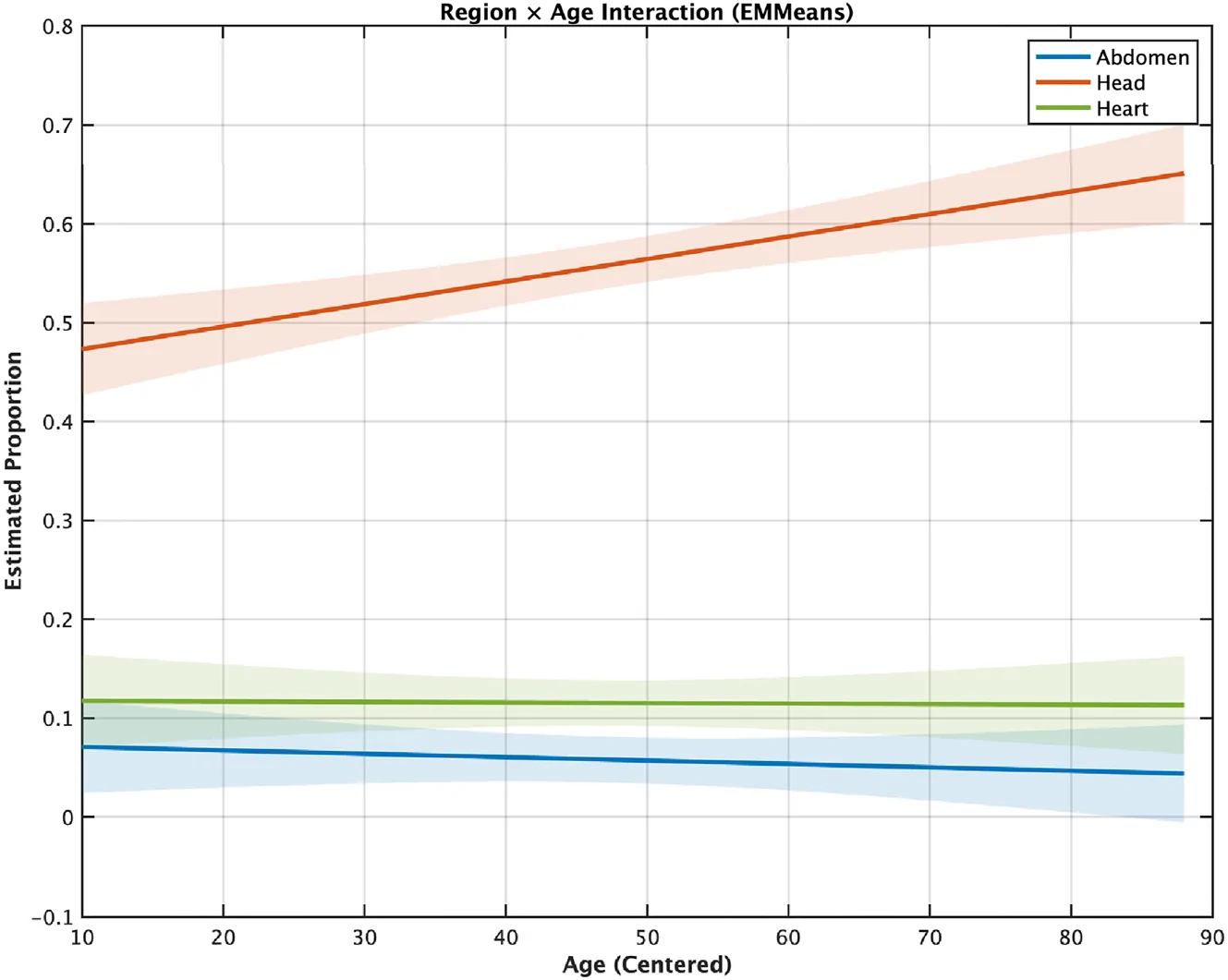
Interaction between body region and age on estimated response proportions. Solid lines represent estimated marginal means (EMMeans) for each body region (head, heart, and abdomen) derived from the linear mixed-effects model (LMM). Shaded areas indicate 95 % confidence intervals. The relative response proportion for the head increased significantly with age, whereas the proportion for the abdomen and heart showed no significant age-related changes.

Simple-slope analyses of age across body regions (Supplementary Table S2_1) showed that the relative proportion of head sensations increased significantly with age (*β*=0.00228, SE=0.000226, *t*(2,737)=10.09, p<0.001), whereas abdominal sensations showed no significant age-related change (*β*=−0.000345, SE=0.000226, *t*(2,737)=−1.53, p=0.127). Cardiac sensations showed a weak downward trend that did not reach statistical significance (*β*=−0.000055, SE=0.000226, *t*(2,737)=−0.24, p=0.808). Trial Order had no significant effect (*F*(1, 16,414)=0.29, p=0.590), confirming that the observed regional shifts across age were robust to task fatigue.

When examining specific chord progressions, the distribution of bodily sensations varied across progressions (Chord × Region interaction: *F*(14, 16,414)=6.79, p<0.001; Figure 4, Supplementary Table S1, S2_2). Simple-effect factor contrasts (Supplementary Table S2_3) showed that progressions with high surprise elicited a significantly higher proportion of cardiac sensations than those with low surprise (*t*(16,414)=3.82, p<0.001). Conversely, progressions with high uncertainty produced a significantly higher relative proportion of head sensations than those with low uncertainty (*t*(16,414)=4.15, p<0.001). Importantly, the age-related shift – marked primarily by an increased proportion of head sensations – remained consistent across chord progression conditions, indicating that the baseline sensory transition with age operates independently of the specific musical context.

**Figure 4:**
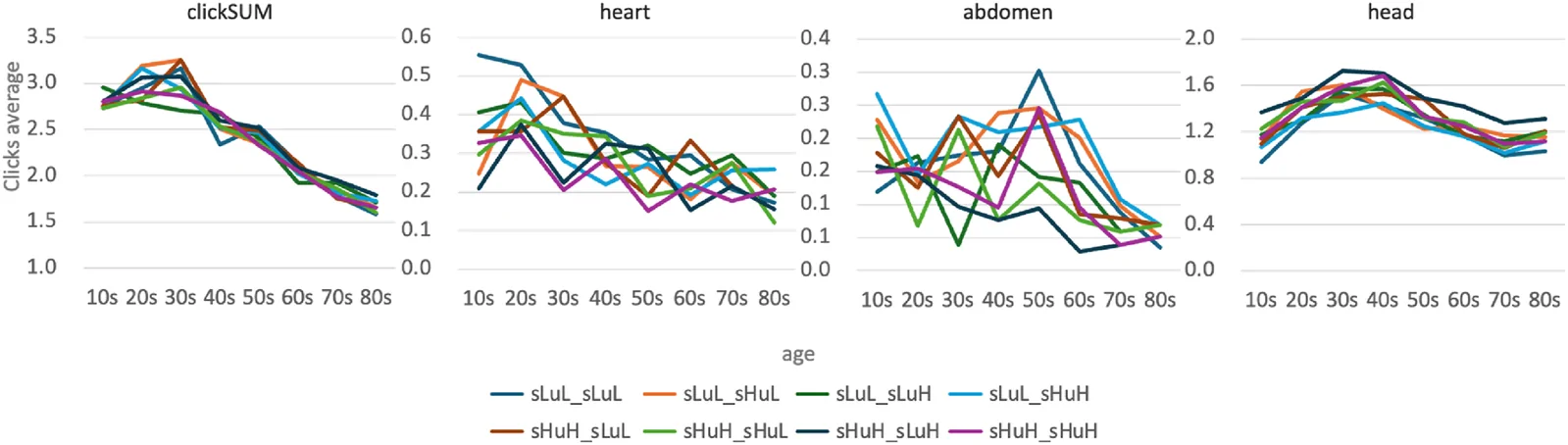
Raw click distributions across age groups for each body region and chord progression. Data represent unadjusted average click counts per chord progression across age groups and body regions. Total raw response volume across the body declined with age after peaking in the 30s, whereas region- and chord-specific variations reflect the underlying unnormalized response density. High-resolution, enlarged body-topography maps for each age group and condition are publicly available on the OSF repository (https://osf.io/wzvqj/).

To test whether the observed age-related sensory shifts followed a non-linear trajectory (e.g., quadratic or inverted U-shaped trends), we fitted an extended linear mixed-effects model incorporating linear (Age) and quadratic (Age^2^) terms (Supplementary Table S3). Controlling for trial presentation order (*F*(1, 16,390)=0.29, p=0.588, *η*_*p*_^*2*^<0.001) and the immediate prior trial response (*F*(1, 16,390)=8,071.09, p<0.001, *η*_*p*_^*2*^=0.330), neither the main effect of quadratic age (*F*(1, 16,390)=2.58, p=0.108, *η*_*p*_^*2*^<0.001) nor non-linear interactions across body regions and chord conditions reached statistical significance (Region × Age^2^: *F*(2, 16,390)=0.72, p=0.487, *η*_*p*_^*2*^<0.001; Chord × Region × Age^2^: *F*(14, 16,390)=1.05, p=0.401, *η*_*p*_^*2*^<0.001). Model comparison indicated that the quadratic term did not significantly improve model fit relative to the linear model (ΔAIC<2), confirming that the age-dependent sensory shifts follow a linear trajectory across adulthood. To verify that these findings were not biased by the proportional transformation or the bounded range of percentage data, we also fit a generalized linear mixed-effects model (GLMM) with a Poisson distribution (Poisson GLMM) (Supplementary Tables S4-1 and S4-2). The GLMM yielded virtually identical patterns, confirming a significant Age × Region interaction (p<0.001) and showing that the age-related shift toward head-centered sensations is robust to model selection.

### Age-group comparisons of bodily sensations across chord progressions

As the LMM analysis, bodily sensations exhibited region-specific linear trends across the lifespan. To further clarify detailed differences between specific age groups (10s to 80s), we conducted post-hoc pairwise comparisons following one-way analysis of variance (ANOVA) for each body region (full statistical test values, adjusted p-values, and effect sizes are available in the public repository; see Data Availability Statement). For whole-body sensations, participants in their 30s reported significantly higher sensation intensity than those in their 60s across multiple chord sequences, including sLuL-sLuL (*W*=−4.13*,* p=0.02, *d*=0.43, 95 % CI [0.16, 0.70]), sLuL-sHuL (*W*=−5.31*,* p=0.001, *d*=0.46, 95 % CI [0.19, 0.74]), sHuH-sLuL (*W*=−3.83*,* p=0.04, *d*=0.39, 95 % CI [0.11, 0.66]), sHuH-sHuL (*W*=−4.59*,* p=0.001, *d*=0.42, 95 % CI [0.15, 0.70]), sHuH-sLuH (*W*=−4.32*,* p=0.01, *d*=0.48, 95 % CI [0.20, 0.75]). Additionally, participants in their 20s showed significantly higher whole-body sensations compared to those in their 60s for the sLuL-sHuH (*W*=−5.18*,* p=0.001, *d*=0.52, 95 % CI [0.24, 0.80]) and sHuH-sHuH sequence (*W*=−4.91*,* p=0.002, *d*=0.41, 95 % CI [0.14, 0.69]). Overall, whole-body sensation intensity peaked during young adulthood (20s–30s) and declined in older age groups (Figures 2 and 4). A notable exception was the sLuL-sLuH sequence, in which sensation intensity was significantly higher not only in the 20s (*W*=−4.32, p=0.010, *d*=0.44, 95 % CI [0.17, 0.72]) and 30s (*W*=−5.30, p=0.001, *d*=0.49, 95 % CI [0.21, 0.77]) but also in the 10s (*W*=−4.82, p=0.001, *d*=0.51, 95 % CI [0.23, 0.79]) relative to those in their 60s (full correlation matrices across all conditions and variables are available in the public repository; see Data Availability Statement).

Regarding cardiac sensations (heart click counts), sensation intensity peaked in the 20s across several chord sequences (Figure 2 and 4). Specifically, participants in their 20s reported significantly higher sensation counts than those in their 60s for the sLuL-sHuL (*W*=−2.98*,* p=0.01, *d*=0.38, 95 % CI [0.11, 0.65]), sLuL-sHuH (*W*=−2.34*,* p=0.03, d=0.33, 95 % CI [0.06, 0.60]), and sHuH-sLuH sequence (*W*=−4.04*,* p=0.01, *d*=0.37, 95 % CI [0.10, 0.65]). Additionally, sensations in the 20s were significantly higher compared to those in the 70s for the sLuL-sLuL (*W*=−2.26*,* p=0.03, *d*=0.31, 95 % CI [0.03, 0.58]), and compared to those in the 50s for sLuL-sLuH (*W*=−2.05*,* p=0.03, *d*=0.12, 95 % CI [−0.15, 0.39]) and the sHuH-sHuL (*W*=−3.14*,* p=0.02, *d*=0.29, 95 % CI [0.01, 0.56]).

In the abdominal region, sensation intensity showed a general tendency to decrease with age. However, we did not identify clear directional changes related to specific chord progressions in the statistical analysis.

For the head sensations, responses exhibited an inverted U-shaped pattern across age groups, peaking in the 30s and 40s (Figure 4). Specifically, sensation intensity for the sLuL-sLuL sequence was significantly higher in individuals in the 30s compared to individuals in the 10s (*W*=4.27*,* p=0.002, *d*=0.42, 95 % CI [0.14, 0.70]) and the 70s (*W*=−3.37*,* p=0.03, *d*=0.40, 95 % CI [0.12, 0.68]). Similarly, the sLuL-sLuH sequence was significantly higher in individuals in the 30s compared to individuals in the 10s (*W*=2.94*,* p=0.03, *d*=0.28, 95 % CI [0.01, 0.56]) and the 70s (*W*=−2.98*,* p=0.01, *d*=0.35, 95 % CI [0.08, 0.63]). Furthermore, the sHuH-sHuL sequence showed significantly higher intensity in the 40s compared to the 10s (*W*=3.01*,* p=0.01, *d*=0.21, 95 % CI [-0.06, 0.48]) and 70s (*W*=−2.74*,* p=0.04, *d*=0.35, 95 % CI [0.07, 0.62]). To confirm whether response speed limits or age-related psychomotor slowing confounded these bodily sensation responses, we first examined trial-level click distributions. Across all 15,698 trials during the 3-second window, the mean click count per trial was 4.64 ± 4.18 (median=2.00, range=1–40), with only a single trial (0.01 %) reaching the maximum count of 40 clicks, indicating no operational ceiling or motor-speed limitations. Furthermore, LMM analysis on relative ROI proportions (Supplementary Table S5) confirmed that the relative proportion of head-centered clicks significantly increased with age (*β*=0.0023, *t*(6,263)=3.55, p<0.001), whereas the relative proportion of heart-centered clicks showed no significant age dependence (*β*=−0.0001, *t*(6,263)=−0.18, p=0.857). Together with the finding that subjective arousal ratings are inherently immune to motor slowing, these results establish that our findings reflect genuine age-related shifts in bodily sensation mapping rather than a motor artifact.

### Relation between age and emotions

To evaluate the impact of musical predictive structure and aging on subjective emotional responses, we fitted linear mixed-effects models (LMMs) separately for Valence and arousal ratings across the eight chord conditions (Supplementary Table S6-1).

For Valence ratings, the omnibus LMM revealed significant main effects of Age (*F*(1, 2,241.87)=6.80, p=0.009, *η*_*p*_^*2*^=0.003) and Chord condition (*F*(7, 5474)=11.54, p<0.001, *η*_*p*_^*2*^=0.015), as well as a significant Age × Chord interaction (*F*(7, 5,474)=3.40, p=0.001, *η*_*p*_^*2*^=0.004). Estimated marginal means (EMMeans; Supplementary Table S6-2) showed that predictable progressions with low surprise and uncertainty at the sequence ending elicited higher pleasantness ratings, with the sLuL-sLuL condition (*M*=5.92, SE=0.06, 95 % CI [5.81, 6.03]) and the sLuL-sHuL condition (*M*=5.82, SE=0.06, 95 % CI [5.71, 5.93]) yielding the highest ratings, whereas progressions ending in high uncertainty were rated lowest (sLuL-sLuH: *M*=5.30, SE=0.06, 95 % CI [5.19, 5.41]). To test whether previous findings in young adults could be replicated in our sample, we ran a subset analysis restricted to participants in their 20s and 30s (n=207), matching the age demographic of our previous study [18]. In this young adult subset, subjective valence ratings showed a significant main effect across musical conditions (*F*(7, 1,442)=10.10, p<0.001), replicating the condition-dependent valence patterns reported in our previous study [18].

For arousal ratings, significant main effects were observed for Age (*F*(1, 2,275.71)=14.76, p<0.001, *η*_*p*_^*2*^=0.006) and Chord condition (*F*(7, 5474)=15.86, p<0.001, *η*_*p*_^*2*^=0.020), along with an Age × Chord interaction (*F*(7, 5,474)=4.40, p<0.001, *η*_*p*_^*2*^=0.006). Progressions with unexpected target transitions elicited higher arousal ratings than fully predictable transitions (e.g., sHuH-sLuH: *M*=5.19, SE=0.06, 95 % CI [5.08, 5.30]; sLuL-sLuH: *M*=4.91, SE=0.06, 95 % CI [4.80, 5.02] vs. sLuL-sLuL: *M*=4.55, SE=0.06, 95 % CI [4.44, 4.66]; sLuL-sHuH: *M*=4.55, SE=0.06, 95 % CI [4.44, 4.66]).

To distinguish true statistical effects from potential model oversensitivity in large samples, we conducted Bayesian model comparisons using BIC approximations (Supplementary Table S7). The main effect of Chord was decisive for both Valence (*BF*_*10*_=2.15 × 10^34^) and Arousal (*BF*_*10*_=2.16 × 10^33^). Conversely, the Age × Chord interaction showed decisive evidence in favor of the null hypothesis for both Valence (*BF*_*01*_=1.34 × 10^8^) and Arousal (*BF*_*01*_=3.96 × 10^6^). Furthermore, the main effect of Age on Valence strongly supported the null model (*BF*_*01*_=73.13), confirming that subjective valence ratings did not systematically vary across age groups. By contrast, the main effect of Age on Arousal yielded only anecdotal evidence (*BF*_*10*_=2.13, *BF*_*01*_=0.47).

As shown in the continuous trajectories in Figure 5, linear mixed-effects modeling and correlation analyses revealed a significant positive trajectory of subjective arousal with age (Figure 5, bottom panel; *ρ*=0.24, p=0.04**).** In contrast, subjective valence ratings showed no age-dependent slope and remained stable across the lifespan (Figure 5, top panel). While model comparisons favored a uniform effect across conditions, post hoc correlation analyses indicated that this positive arousal trajectory was most distinctly observed in predictable musical structures, including sLuL-sLuL (*ρ*=0.29, p=0.01) and sHuH-sLuL (*ρ*=0.32, p=0.003). To complement these continuous patterns, categorical Kruskal-Wallis tests across age bins confirmed significant differences in arousal for sLuL-sLuL (*χ* ^*2*^=17.08, p=0.02, *ε* ^*2*^=0.02) and sHuH-sLuL (*χ* ^*2*^=17.41, p=0.02, *ε* ^*2*^=0.02). Pairwise comparisons revealed that individuals in their 80s reported significantly higher arousal ratings for sLuL-sLuL than those in their 10s (*W*=4.40, p=0.001, *d*=0.56, 95 % CI [0.23, 0.89]) and 20s (*W*=4.31, p=0.003, *d*=0.50, 95 % CI [0.17, 0.83]), and for sHuH-sLuL relative to their 10s (*W*=3.98, p=0.01, *d*=0.47, 95 % CI [0.15, 0.80]) and 20s (*W*=4.43, p=0.001, *d*=0.51, 95 % CI [0.19, 0.84]) (for full correlation matrices and pairwise test statistics, see Data Availability Statement).

**Figure 5:**
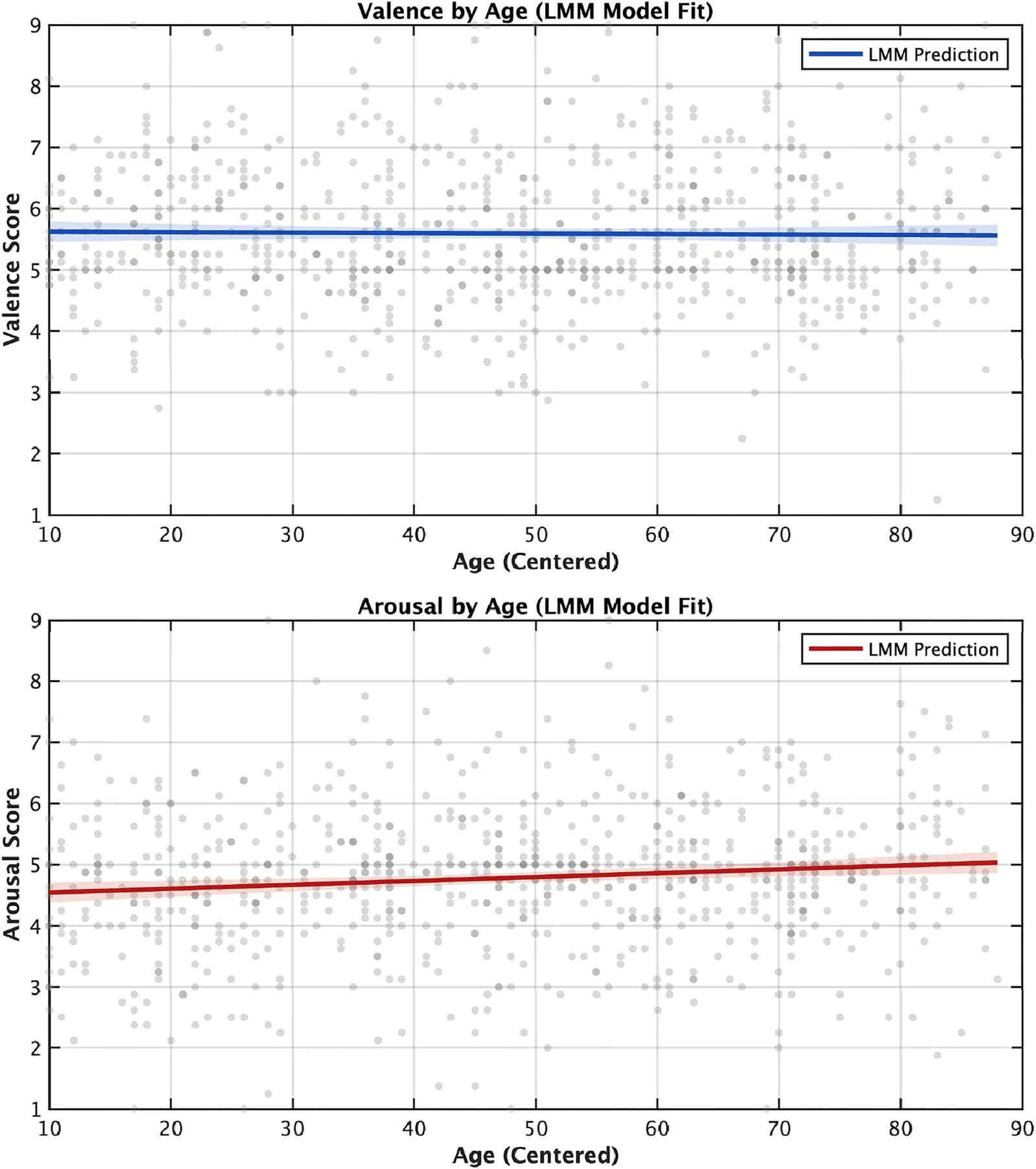
Age-related trajectories for emotional valence and arousal scores. Scatter points denote individual participant mean ratings (n=784). Solid lines and shaded areas represent the fixed-effect regression trajectories and corresponding 95 % confidence intervals estimated by linear mixed-effects models (LMMs). Emotional valence did not change significantly across age groups, whereas arousal increased significantly with age.

## Discussion

In this study, we aimed to clarify the differences in interoception and bodily sensations experienced while listening to music throughout the lifespan. We hypothesized that interoceptive and bodily sensations experienced while listening to music vary with age and exhibit region-specific developmental trajectories. Our results partially supported this hypothesis. Although the continuous main effect of age in the LMM did not capture an overall linear decline, nonparametric analyses revealed significant age-related differences across age groups, with overall bodily sensation intensity peaking in young adulthood (20s–30s) and declining thereafter. This age-related decline was particularly evident for highly predictable sounds with low uncertainty and low prediction error, whereas sensations localized in the head did not show a linear decline with age. Furthermore, LMM analyses demonstrated a significant Region × Age interaction, driven by a continuous increase in the relative proportion of head-centered sensations. In contrast, non-parametric age-group comparisons revealed distinct non-linear trajectories across regions; Specifically, sensations in the chest peaked in the 20s and then decreased, as confirmed by post-hoc comparisons. In the abdomen, we observed a general decreasing trend, although pairwise age-group differences did not reach significance. In contrast, head sensations exhibited a distinct pattern depending on the analytical metric: because total bodily sensation counts declined across the lifespan, the relative proportion of head sensations showed a significant linear increase with age. Conversely, post hoc comparisons of raw click counts revealed a middle-adulthood peak (30s–40s) across several chord conditions.

Regarding emotional ratings, the LMM and Bayesian model comparisons provided nuanced insights into the effects of musical structure and age. The main effect of Chord was significant for both Valence and Arousal, confirming that predictable progressions (low surprise and low uncertainty) consistently elicited higher valence, whereas unexpected transitions evoked higher arousal. Crucially, although LMM omnibus tests indicated significant Age × Chord interactions, Bayesian comparisons provided decisive evidence in favor of the null interaction hypothesis for both Valence (*BF*_*01*_=1.34 × 10^8^) and Arousal (*BF*_*01*_=3.96 × 10^6^). Furthermore, the main effect of Age on Valence strongly supported the null hypothesis (*BF*_*01*_=73.13), indicating that subjective pleasantness remains stable across the lifespan. In contrast, arousal ratings showed a positive, continuous correlation with age (*ρ*=0.24) – an overall trend that was notably pronounced in post hoc analyses for highly predictable structures (sLuL-sLuL and sHuH-sLuL), with older adults (80s) reporting significantly higher arousal than younger groups.

Humans inherently integrate top-down stimulus predictability with bottom-up interoceptive sensations [29], [30], [31], [32], [33], [34], [35]. Awareness of these bottom-up interoceptive sensations requires sensitivity to interoceptive signals transmitted through various sensors, such as mechanoreceptors and chemoreceptors, which connect the peripheral and central nervous systems along the axes of the heart, respiratory system, and stomach [36].

According to a review by Murphy and colleagues [37], interoceptive ability, as assessed by a heartbeat discrimination task, has been shown to perform comparably in children (ages 6–11) and adults [38]. Although research is limited, these abilities are thought to develop during childhood. Studies using the heartbeat tracking task in children have shown that interoceptive accuracy (IAc) tends to increase with age [39]. Similarly, assessments using the Self-Awareness Questionnaire (SAQ) have indicated that children’s interoceptive sensibility also improves as they grow older [40]. However, studies indicate that interoceptive abilities, such as exteroception and proprioception, decline with age due to reduced sensitivity in the central nervous system and peripheral receptors [41].

Consistent with previous research demonstrating an age-related decline in interoceptive ability [41], our results indicate that bodily responses to auditory stimuli generally peak during young adulthood (20s–30s) and diminish with age. Notably, this decline was more pronounced for stimuli that were highly predictable and consistent with prior expectations – that is, stimuli associated with low prediction error and low uncertainty.

Previous research on age-related differences in top-down stimulus predictability indicates that children, due to their limited past experience, place greater emphasis on the novelty of incoming stimuli and rely more on episodic memory when processing new information. In contrast, as the brain matures beyond young adulthood, individuals develop and maintain more refined predictive models. Older adults, in particular, are shown to rely significantly on their extensive knowledge base and long-term semantic memory representations acquired over a lifetime, leading to a greater reliance on top-down predictions [42], [43], [44].

In this study, we observed a notable dissociation: subjective arousal exhibited an overall age-related increase – most clearly detected in post hoc evaluations of predictable chord sequences (sLuL-sLuL and sHuH-sLuL), whereas counts of bodily sensations in interoceptive regions decreased. Rather than attributing this pattern directly to altered predictive error weighting, it aligns with findings from Saito et al. [45], who reported an age-related reduction in physiological-emotional coherence among older adults. Older individuals may maintain or increase subjective emotional arousal through top-down cognitive and semantic appraisal, even as peripheral bodily reactivity diminishes.

Regarding valence, Bayesian evidence strongly supported no systematic variation in subjective valence ratings across age groups (*BF*_*01*_=73.13). Crucially, to clarify the discrepancy with previous literature, we conducted a targeted subset analysis limited to young adults (20s–30s). This subset analysis replicated our previous research [18], confirming that the effect of musical expectation on valence ratings is robust among young adults. Taken together, these findings suggest that although peripheral bodily reactivity directly informs valence appraisal in early adulthood, overall valence appraisal across the full lifespan remains preserved despite the age-related decline in bodily sensation magnitude. This indicates that age-related changes in bodily reactivity do not directly diminish subjective hedonic appreciation of musical harmony.

This study has several limitations. From the perspective of experimental methods, first, the experiments were conducted online, which may have reduced the experimental control compared to a laboratory setting. Nevertheless, because our objective was to understand how music shapes affective experiences in everyday life and lifestyle contexts, capturing responses outside traditional laboratory settings provides valuable ecological validity. Second, we did not account for the potential influence of factors such as antihypertensive medications, which are commonly prescribed in older adulthood and may affect bodily responses [46]. However, these unmeasured factors alone cannot account for our main conclusions, because our paradigm evaluated within-subject differences across distinct musical conditions and revealed highly region-specific trajectories. Nonetheless, future laboratory-based studies should systematically record health status and medication history. A third limitation is that this study focused exclusively on comparing eight musical chord conditions and did not include a non-music (silent) control condition. As a result, it remains unclear whether the observed age-related patterns in body region selection are specific responses to musical stimuli or reflect more general bodily awareness. Future studies should consider including a silent condition or emotionally neutral tasks to better isolate the specific contribution of emotional musical stimuli to bodily sensation mapping.

A further limitation concerns the interpretability of the bodily sensation maps themselves. Although participants were instructed to report actual bodily sensations in response to chord sequences, it remains possible that some responses were influenced by symbolic or culturally learned associations (e.g., associating the heart with love or positive emotions). While younger participants showed distinct spatial mapping patterns, interpreting these as purely abstract or metaphorical associations warrants caution. Cognitive developmental research indicates that the comprehension of metaphors and figurative concepts continues to develop throughout childhood and adolescence, extending into adulthood [47]. Therefore, rather than reflecting heightened abstract or metaphorical reasoning skills, the age-related differences in body sensation maps may reflect differences in sensorimotor integration, bodily self-awareness, or reporting styles across the lifespan. Moreover, the degree to which individuals can consistently localize emotions in specific body parts likely varies across participants. Given this variability, the association between emotion and bodily regions should be interpreted with caution. Furthermore, based on our previous review of body map studies [48], we have observed that many bodily sensation maps align with established patterns of bottom-up physiological arousal, such as increases in heart rate or visceral sensations. In addition, prior research [13], 14] has shown that body maps associated with emotions exhibit similar patterns across different cultural groups, suggesting a certain degree of universality and a basis in embodied emotional experience. Nonetheless, to account for individual variability in reporting and to objectively validate whether reported locations directly correlate with autonomic activity, future research should incorporate direct physiological recordings (e.g., electrocardiography or galvanic skin response) in conjunction with body mapping.

Furthermore, the observed patterns in self-reported body maps warrant careful interpretation of their underlying mechanisms. Because self-reports capture multidimensional affective awareness – integrating top-down cognitive appraisal with bottom-up somatic signals – they cannot isolate pure physiological reactivity or establish direct causal links between bodily sensations and affective dimensions across age cohorts. To enhance interpretative validity, future studies should adopt multi-dimensional approaches that include behavioral and neurophysiological measures.

Another important limitation of this study concerns the differential age-related patterns observed across specific body regions. While sensations in interoceptive regions (chest and abdomen) showed only subtle, non-significant downward trends – partially differing from well-documented age-related reductions in interoceptive sensitivity [49], 50] – head-related sensations exhibited a non-monotonic pattern, increasing in middle adulthood (30s–40s) in post hoc comparisons, consistent with the maturation of executive functions [51]. However, because our study relies on a cross-sectional design, these regional patterns must be interpreted cautiously, as they cannot definitively distinguish between intra-individual developmental trajectories and cohort-specific effects. Future research should adopt longitudinal and neurophysiological approaches that consider the functional characteristics of each body region and age-related changes to further clarify the mechanisms underlying these developmental trajectories.

Finally, another limitation concerns the treatment of age as a categorical variable using 10-year intervals. This decadal classification was determined a priori concerning both theoretical and empirical frameworks in developmental psychology – particularly Erikson’s psychosocial developmental stages [52] – and is commonly adopted in lifespan and aging research (e.g. [53], [54], [55]). While LMM sensitivity analyses using Age as a continuous predictor confirmed the consistency of our main findings, using 10-year bins may reduce resolution and potentially obscure fine-grained non-linear shifts. Future studies should consider alternative continuous or narrow-bin grouping strategies to better capture nuanced developmental trajectories across the lifespan.

## Conclusions

This study demonstrates that, although overall bodily sensation intensity peaks non-linearly in young adulthood (20s–30s) and then declines with age, relative head-centered bodily sensations increase significantly across the adult lifespan, whereas bottom-up interoceptive sensations (chest and abdomen) show no significant linear changes. Importantly, subjective valence appraisal remains preserved across generations, while subjective arousal increases with age, particularly pronounced in evaluations of predictable musical structures through top-down stimulus prediction. Building on our previous findings in young adults [18], these lifespan results reveal how the dynamic interplay among predictive mechanisms, somatic sensations, and emotional appraisal evolves across age cohorts. These findings may deepen our understanding of how the relationship between predictive mechanisms and physical responses evolves across the lifespan. They may also provide a scientific basis for identifying prediction-based music that promotes affective engagement and emotional regulation while accounting for generational differences in physical and mental characteristics.

## Supplementary Material

Supplementary Material
