# Supplementary material for: Lifetime changes in body maps investigated with music
Source: Transl Neurosci. 2026 Oct 23;17(1):20250407. doi: 10.1515/tnsci-2025-0407 (PMC13693815; doi:10.1515/tnsci-2025-0407)
Supplement: Supplementary file 1 [file j_tnsci-2025-0407_suppl_001.docx]

Figure S1. Sample size and sex distribution across age groups. Bar graph showing the total sample size (*N* = 784) and sex distribution (Female: orange, *n* = 372; Male / Unspecified: blue, *n* = 412) categorized into eight 10-year age groups (10s to 80s).

Figure S2. Experimental task interface and body mapping outline. Example of body map clicks (top) and user interface screens for the experiment (A–C). (A) Preliminary audio verification task to set a comfortable volume and verify sound playback. (B) Main body mapping task screen that provides task instructions and the body outline for clicking felt bodily sensations within 3 seconds. (C) Emotional and cognitive evaluation screen using 9-point Likert scales for valence, arousal, creativity, and beauty.

A.

B.

C.

Figure S3. Clicked body positions for each chord progression within each age group. Visualization of individual click distributions on the body outline across eight distinct chord progressions for each of the eight age groups (10s to 80s).

Table S1. Linear Mixed Model for Proportions Controlling for Lagged and Order Effects

Note. Linear mixed-effects model controlling for lagged and order effects. *N* = 16,464 observations across 784 participants. Continuous covariates were mean-centered. Degrees of freedom (df_1_, df_2_) were calculated using Satterthwaite’s approximation.

Table S2. Linear Mixed Model Simple Effect Analysis

Table S2_1. Simple Effects of Age across Regions

1-1. ANOVA for Simple Effects of Age

1-2. Parameter Estimates for Age Slopes

Note. Simple effects of age across body regions. Parameter estimates (β) indicate proportion change per 1 year of age. Degrees of freedom (df) were calculated using Satterthwaite’s approximation. *** *p* < 0.001, n.s. *p* > 0.05. CIs represent 95% confidence intervals.

Table S2_2. Estimated Marginal Means for Region × Chord Interaction (EMMeans)

Note. Estimated Marginal Means (EMMeans), Standard Errors (SE), and 95% Confidence Intervals (CIs) for Region × Chord interaction. Degrees of freedom (df) were calculated using Satterthwaite’s approximation.

Table S2_3. Simple Effects of Chord Condition and Factor Contrasts within Regions

3-1. ANOVA for Simple Effects of Chord across Body Regions

3-2. Planned Factor Contrasts for High vs. Low Surprise and Uncertainty across Regions

Note. (3-1) Simple effects of chord condition within each body region. (3-2) Planned factor contrasts comparing High vs. Low levels of Surprise and Uncertainty across body regions (Heart, Head, Abdomen). Degrees of freedom (df = 16414) were calculated using Satterthwaite’s approximation. Continuous covariates were mean-centered. CIs represent 95% confidence intervals. *** *p* < 0.001, n.s. *p* > 0.05.

Table S3. Linear Mixed Model Analysis Incorporating Linear and Quadratic Age Effects

Note. Linear mixed-effects model incorporating linear and quadratic age effects. Continuous predictors were mean-centered. Degrees of freedom (df) were calculated using Satterthwaite’s approximation.

Table S4. Generalized Linear Mixed-Effects Model (GLMM) Sensitivity Analysis for Bodily Sensation Counts

Table S4-1. Standard Poisson GLMM Omnibus Tests

Note. Generalized linear mixed-effects model (GLMM) with Poisson distribution evaluating unadjusted raw click counts. *df_1_* and *df_2_* represent numerator and denominator degrees of freedom, respectively. *** *p* < 0.001, n.s. *p* ≥ 0.05.

Table S4-2. Poisson GLMM with Observation-Level Random Effects (OLRE) Omnibus Tests

Note. Generalized linear mixed-effects models (GLMM) with Poisson GLMM. Table S4-1 displays the standard Poisson model, while Table S4-2 incorporates Observation-Level Random Effects (OLRE) to account for potential overdispersion. Both models confirm consistent significant main effects of Chord and Region, as well as significant Age × Region and Chord × Region interactions. *** *p* < 0.001, n.s. *p* ≥ 0.05.

Table S5. Linear Mixed Model Analysis on Relative ROI Proportions

Table S5-1. Parameter Estimates for Heart Region Proportions

Table S5-2. Parameter Estimates for Head Region Proportions

Note. Fixed-effects parameter estimates from linear mixed-effects models (LMM) predicting relative click proportions in Heart (S5-1) and Head (S5-2) regions. N = 6,272 observations across 784 participants. Continuous predictor Age was centered. The reference category for chord progression conditions is sLuL-sLuL. Degrees of freedom (df) were estimated using Satterthwaite's approximation. CI = Confidence Interval; SE = Standard Error. * *p* < 0.05, ***p* < 0.01, *** *p* < 0.001.

Table S6. Linear Mixed Model Analysis of Valence and Arousal Ratings

Table S6-1. Type III ANOVA Tables for Linear Mixed-Effects Models with Partial Eta-Squared (*η_p_^2^*)

Note. Type III ANOVA table for linear mixed-effects models fitted using REML with Satterthwaite’s approximation for degrees of freedom (df_1_, df_2_). Partial Eta-Squared (*η_p_^2^*) was calculated as (F x df_1_) / (F x df_1_ + df_2_). *N* = 6,272 observations across 784 participants.

Table S6-2. Estimated Marginal Means (EMMeans) and 95% Confidence Intervals

Note. Estimated Marginal Means (EMMeans), Standard Errors (SE), and 95% Confidence Intervals (CIs) derived from linear mixed-effects models fitted using REML. Participant age was mean-centered. Degrees of freedom (df) were calculated using Satterthwaite’s approximation.

Table S7. Bayesian Model Comparison & Bayes Factors (*BF_10_* and *BF_01_*)

Note. Bayesian model comparison using Bayesian Information Criterion (BIC) approximations for linear mixed-effects models fitted via ML. Bayes factors were calculated as *BF_01_* = exp((*BIC_H1_* - *BIC_H0_*) / 2) and *BF_10_* = 1 / *BF_01_*. *BF_01_* > 100 indicates decisive evidence for *H_0_*, *while BF_10_ >* 100 indicates decisive/extreme evidence for *H_1_* according to Jeffreys’ benchmarks.
